# Cardiogenic shock caused by Takotsubo syndrome complicated with severe anxiety: A case report and literature review

**DOI:** 10.1097/MD.0000000000027812

**Published:** 2021-11-12

**Authors:** Shu Fang, Yu Wang, Peng-Kang He, Xiao-Ning Han, Ying Yang, Tao Hong, Yan-Jun Gong

**Affiliations:** a Department of Cardiology, Peking University First Hospital, Beijing, China; b Department of Endocrinology, Peking University First Hospital, Beijing, China.

**Keywords:** cancer, cardiogenic shock, death, mental disorder, Takotsubo syndrome

## Abstract

**Rationale:**

Takotsubo syndrome (TTS) is characterized by transient and reversible left ventricular systolic dysfunction, which are often associated with acute physical or emotional stressors. Cancer is one of the comorbidities in TTS, and TTS is even considered as a paraneoplastic syndrome, but its mechanism remains unclear. We report a patient in whom cancer and untreated mental disorders triggered TTS.

**Patient concerns:**

A 59-year-old man was transferred to the Department of Cardiology because of acute onset of severe chest pain and dyspnea before cystoscopy. He presented with hematuria, had been diagnosed with a high-grade urothelial bladder cancer, and underwent transurethral resection of bladder tumors 4 months previously. He had severe anxiety regarding recurrence and death from cancer, especially after the hematuria recurred.

**Diagnosis:**

TTS and severe anxiety.

**Interventions:**

The results of coronary angiography, a left ventriculogram, echocardiography, and the clinical outcome led to the diagnosis of TTS. The patient was treated with extracorporeal membrane oxygenation support, mechanical ventilation, and drugs for heart failure and anxiety.

**Outcomes:**

Echocardiography showed normal wall motion on day 6 of symptom onset. Six months after symptom onset, the anxiety score was reduced from 12 to 11, and the patient had no episodes of any discomfort, and no evidence of cancer recurrence was observed.

**Lessons:**

Patients with cancer and TTS have a higher level of stress, and physicians need to pay more attention to early screening and early treatment of mental disorders in these patients. Prompt and effective multidisciplinary treatment, including psychological counseling and antianxiety drugs, can improve the prognosis in such cases.

## 1 Introduction

Takotsubo syndrome (TTS) is a rare cardiovascular disease (CVD), characterized by transient and reversible left ventricular systolic dysfunction, and nearly half of these patients have neurological or psychiatric disorders.^[1,2]^ TTS often mimics ST-segment elevation myocardial infarction (MI) or non-ST-segment elevation MI without evident coronary artery stenosis.^[3,4]^ Mental disorders are reported in 30% to 50% of patients with cancer, and they have a low rate of receiving psychological support and prefer to manage symptoms by themselves.^[5]^ Patients with cancer and mental disorders have an increased risk of cancer-specific mortality.^[6]^ CVD and cancer continue to be the leading causes of death worldwide, but the relationship between cancer and CVD remains unclear.^[7]^

Studies have shown a higher incidence of TTS in patients with cancer and they have an increased risk of adverse events.^[1,4,8]^ Cancer and TTS share common risk factors, such as an older population, stress, and physical stressors.^[1,8,9]^ We report a case of cardiogenic shock caused by TTS due to a fear of tumor recurrence and review the current research on the relationship between TTS and cancer.

## 2 Case presentation

A 59-year-old Chinese man was transferred from the Department of Urology to the Department of Cardiology owing to sudden severe chest pain and dyspnea before cystoscopy. He denied the habit of smoking tobacco, and had chronic arterial hypertension and diabetes. Eight months before the index hospitalization, the patient had been diagnosed with high-grade urothelial bladder cancer (T1, G2) and presented with recurrent hematuria. He underwent transurethral resection of bladder tumors 4 months previously followed by regular intravesical immunotherapy with Bacillus Calmette–Guérin. He did not complain of any discomfort or symptoms, until hematuria recurred before admission.

**Figure 1:**
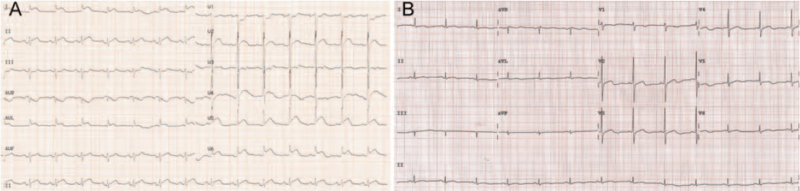
Electrocardiographic changes in the patient. ST-segment elevation in leads I, II, aVL, and V4-6 was observed at the onset of Takotsubo syndrome (A), followed by inverted T waves or biphasic T waves in leads I, II, aVL, and V2-6 on day 11 after onset (B).

**Figure 2:**
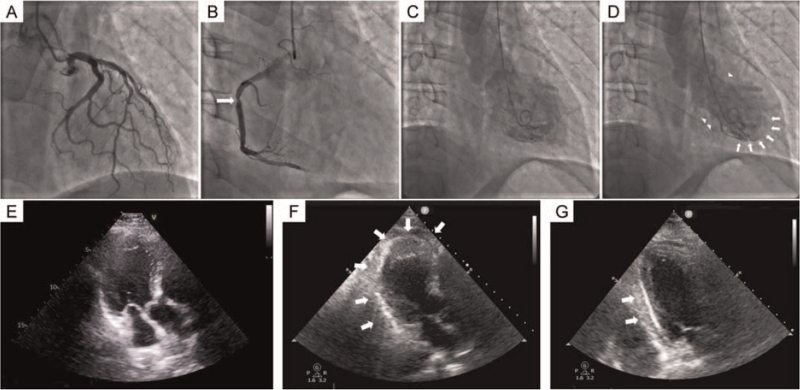
Representative changes of Takotsubo syndrome in the patient. Coronary angiography shows minor atherosclerosis in the left anterior descending artery and circumflex branch of the left coronary artery (A). Slow flow was observed in the right coronary artery, and 50% stenosis (arrow) was observed in the middle right coronary artery (B). Left ventriculography shows normal wall motion during diastole (C), but apical akinesia (arrow) with basal hyperkinesis (triangle) during systole (D). Normal echocardiography at admission (E). Bedside echocardiography shows akinesia of the apex and the left ventricular posterior wall (arrow) at the onset of Takotsubo syndrome (F). On day 4 after onset, only slight akinesia in the mid-segment of the left ventricular posterior wall (arrow) with a normal ejection fraction of 70% (G) can be seen.

Vital signs were a remarkably elevated blood pressure of 200/120 mm Hg, heart rate of 101 beats/min, respiratory rate of 26 breaths/min, and oxygen saturation of 92% in room air. Electrocardiography showed ST-segment elevation in leads I, II, aVL, and V4-6 (Fig. 1A). After urgent consultant with a cardiologist, the patient was suspected as having acute MI and was subject to cardiac catheterization. Cardiac catheterization showed moderate atherosclerotic changes and slow flow in the coronary arteries (Fig. 2A, B). Left ventriculography conducted at the same time showed apical akinesia with basal hyperkinesis, and the ejection fraction was 30% (Fig. 2C, D). As the catheter was pulled back from the left ventricle to the aorta, left ventricular outflow tract obstruction was not observed. The patient was then sent to the cardiac care unit (CCU) because of severe heart failure.

On the day of admission to the Department of Urology, no positive findings were observed in laboratory values and echocardiography (Fig. 2E). However, emergency laboratory testing at symptom onset showed elevated concentrations of creatine kinase isoenzymes (12.4 ng/mL; normal: <5 ng/mL), troponin I (1.295 ng/mL; normal: <0.03 ng/mL), and D-dimer (1.12 mg/L; normal: <0.24 mg/L). Concentrations of B-type natriuretic peptide (12 pg/ml; normal: <100 pg/mL) and C-reactive protein (1.63 mg/L; normal: <3 mg/L) were normal. Further work-up showed normal concentrations of circulating catecholamines, autoimmune antibody, and thyroid hormone. Bedside echocardiography at symptom onset showed akinesia in the apex and in the left ventricular posterior wall (Fig. 2F).

The patient rapidly developed cardiogenic shock after being sent to the CCU. Despite receiving high-dose dopamine and metaraminol, the patient still had marked hypotension of approximately 65/45 mm Hg and loss of consciousness. The patient urgently had extracorporeal membrane oxygenation support and mechanical ventilation performed, which rapidly improved his hemodynamics. On day 4 of symptom onset, the patient's ejection fraction had recovered (70%) as shown by an echocardiographic examination, with only slight akinesia in the mid-segment of the left ventricular posterior wall (Fig. 2G). Therefore, extracorporeal membrane oxygenation was removed and nasal duct ventilation was gradually resumed. On day 6 of symptom onset, echocardiography showed complete recovery of wall motion abnormalities. Additionally, the highest concentration of troponin I was 3.146 ng/mL, which did not correspond to the severity of cardiogenic shock caused by MI. Therefore, TTS was diagnosed on the basis of Heart Failure Association of the European Society of Cardiology diagnostic criteria.^[10]^

During hospitalization, on questioning the patient regarding recent stressors, he reported that he always pretended to be strong in front of his family after the diagnosis of cancer and never wanted to talk about this disease with his family. He often felt lonely, nervous, and could not sleep. He was particularly concerned that the cancer would recur because of the recurrence of hematuria, and he always cried out during the CCU stay because he was afraid of dying immediately. The Hospital Anxiety and Depression Scale was applied, and the score for anxiety was 12 and that for depression was 3. Because of the high level of anxiety, the patient was treated with estazolam and lorazepam as advised by a psychiatrist. Psychological counseling was also suggested.

On discharge, a widespread T wave change in electrocardiography was still observed without ST-segment elevation (Fig. 1B). The following treatment was recommended: aspirin 100 mg once daily, sacubitril valsartan sodium 100 mg twice daily, metoprolol tartrate 25 mg twice daily, atorvastatin 20 mg once daily, estazolam 1 mg every night, and lorazepam 0.25 mg once daily. At a recent follow-up 6 months after onset, the score for anxiety was reduced to 11, and the patient had no episodes of chest pain or dyspnea, and no evidence of cancer recurrence was noted.

## 3 Discussion

TTS is an acute cardiovascular disorder, accounting for approximately 2% to 3% of patients with acute coronary syndrome (ACS).^[3]^ The rate of death from any cause of TTS is 5.6%/patient-years, and a significant percentage of in-hospital complications is comparable with that for ACS.^[1]^ TTS frequently recurs, with an average recurrence rate of 2% to 4% each year and up to 20% at 10 years.^[11,12]^ TTS is more prevalent in postmenopausal women, and Asian patients are more likely to suffer from a serious condition (cardiogenic shock) than Europeans.^[2]^ The etiologies in patients with TTS remain unclear, but sudden and extreme stress resulting in abnormal myocardial, coronary, systemic vascular, adrenal, hypothalamic–pituitary–adrenal axis, and neural responses are widely accepted.^[3,13]^ The symptoms and biomarker levels of TTS may resemble those of ACS. Therefore, physicians have a difficult challenge in making a differential diagnosis, and coronary angiography is mandatory. The current treatment strategies in TTS aim to reduce life-threatening complications with supportive therapy and wait for spontaneous recovery.^[3]^ Angiotensin converting-enzyme inhibitors or angiotensin II receptor blockers improve survival at 1 year of follow-up. β-blockers might be useful in those with persistent anxiety, an elevated sympathetic tone, or recurrent episodes.^[12]^ Psychological counseling or antianxiety drugs may be beneficial for preventing recurrence of TTS and improving long-term outcomes for patients who are mainly triggered by emotional stressors or suffering from neuropsychiatric disorders.^[3]^

Cancer is a comorbidity that is commonly found in TTS, either in history or as an active neoplasm,^[1,8,9,14]^ which has led to TTS being called a paraneoplastic syndrome. In the largest published registry on TTS, cancer was found in 16.6% of 1750 patients with TTS.^[1]^ Over a 6-year follow-up, the incidence rate of TTS was still up to 11% in 275 patients with cancer.^[9]^ Patients with TTS show a worse outcome when complicated by cancer. A previous study reported a higher in-hospital and long-term mortality in patients with TTS and cancer than in those with TTS without cancer.^[14]^ A meta-analysis on patients with TTS and cancer showed a 3.33-fold incidence of adverse events occurring in hospital (including life-threatening arrhythmias, cardiogenic shock, thromboembolism, and requirement for respiratory support) and a 2.08-fold incidence at follow-up (including all-cause mortality and re-hospitalization for cardiovascular disease).^[8,15]^

Previous studies have reported that the diagnosis of cancer may decrease the psychic threshold for stress stimuli,^[4]^ and nearly 50% of patients with cancer suffer from significant emotional disorders and physical stressors, which might increases the effect of further external stressors.^[8,16]^ Patients with cancer and mental disorders have longer hospitalizations and a worse prognosis than those without mental disorders, but often remain unnoticed during somatic treatment settings.^[5,17]^ Patients with cancer prefer to manage psychological difficulties by themselves. A review reported that 19% of patients with cancer did not know the availability of psychological support, 13% thought psychological treatment was ineffective, and 10% were afraid of stigmatization.^[18]^ In a recent study, men and patients aged 65 years or older had the lowest probability of receiving a psychological consultation.^[5]^

The causes of TTS in patients with cancer are multifactorial. Patients with TTS and cancer are associated with a greater chance of developing endothelial dysfunction in epicardial and microvascular coronary arteries compared with non-cancer patients. Additionally, patients with TTS and cancer are associated with the diagnosis of neoplastic disease, a lower threshold for stress stimuli, as well as the stress associated with its treatment, pain in the course of the disease, and complications of operative therapy or chemotherapy.^[4,14,19,20]^ Moreover, cardiac adrenoreceptor sensitivity is increased in patients with cancer owing to paraneoplastic mediators and a chronic inflammatory state. A chronic inflammatory state is caused by excess levels of inflammatory mediators (e.g., cytokines, free radicals, prostaglandins, catecholamines, and growth factors) and also plays an important role in the etiology of CVD.^[4,19,21]^ CVD and cancer share common pathogenic pathways. The disease itself causes abnormal physiological changes, and psychological and physical stress caused by these 2 diseases also aggravate the primary disease because of a chronic and recurrent course.^[7]^

In this case, the common causes of TTS were excluded, including catecholamines (normal concentrations), neurological diseases (negative symptoms and normal neurological physical examination), and drugs (no special medical therapy). Therefore, emotional stress and cancer were considered as the cause of TTS. Our patient experienced a difficult psychological condition during the past year because of urothelial bladder cancer, and this represented a milieu for developing TTS. When further elements of psychological distress (fear of cancer recurrence due to hematuria) occurred in this patient's life, the situation of a decreased psychic threshold for stress stimuli and physical and psychological exhaustion contributed to trigger TTS and the following cardiogenic shock. Notably, normal circulating catecholamine concentrations and normal inflammatory mediators at the onset of TTS indicated a higher sensitivity of cardiac adrenoreceptors in this patient. Survivors of cancer experience long-term psychological difficulty.^[22]^ Therefore, unsurprisingly, the anxiety scores in our patient were not greatly decreased at follow-up, which highlights the importance of long-term follow-up care. Additionally, Hospital Anxiety and Depression Scale anxiety scores might be not sufficient to evaluate the acute changes in emotional stress at the onset of TTS.

## 4 Conclusion

In recent cancer statistics, 1 in 5 people will develop cancer in their lifetime. Therefore, a considerable number of patients with cancer may present with clinical signs of TTS or TTS may be the first presentation of cancer as a paraneoplastic event.^[23]^ A triple hit of cancer, its treatment, and development of TTS require good supportive care and a multidisciplinary approach. Additionally, screening for mental disorders in patients with cancer as part of routine treatment is required.

## Author contributions

**Conceptualization:** Peng-Kang He, Xiao-Ning Han, Ying Yang, and Tao Hong.

**Data curation:** Shu Fang and Yu Wang.

**Supervision:** Tao Hong and Yan-Jun Gong.

**Writing - original draft:** Shu Fang.

**Writing - review & editing:** Yan-Jun Gong.
